# Gamification in Mobile Apps for Children With Disabilities: Scoping Review

**DOI:** 10.2196/49029

**Published:** 2024-09-06

**Authors:** Ebrahim Mahmoudi, Paul Yejong Yoo, Ananya Chandra, Roberta Cardoso, Carlos Denner Dos Santos, Annette Majnemer, Keiko Shikako

**Affiliations:** 1 School of Physical and Occupational Therapy McGill University Montreal, QC Canada; 2 Center for Interdisciplinary Research in Rehabilitation of Greater Montreal (CRIR) Montreal, QC Canada; 3 Division of Neurosciences and Mental Health The Hospital for Sick Children Toronto, ON Canada; 4 McGill University Health Center Research Institute Montreal, QC Canada; 5 Département de systèmes d'information et méthodes quantitatives de l’École de gestion de l’Université de Sherbrooke Sherbrooke, QC Canada

**Keywords:** mobile health, mHealth, gamification, children with disabilities, mobile phone

## Abstract

**Background:**

Children with disabilities face numerous challenges in accessing health services. Mobile health is an emerging field that could significantly reduce health inequities by providing more accessible services. Many mobile apps incorporate gamification elements such as feedback, points, and stories to increase engagement and motivation; however, little is known about how gamification has been incorporated in mobile apps for children with disabilities.

**Objective:**

This scoping review aims to identify and synthesize the existing research evidence on the use of gamification in mobile apps for children with disabilities. Specifically, the objectives were to (1) identify the categories of these mobile apps (eg, treatment and educational) (2), describe the health-related outcomes they target, (3) assess the types and levels of gamification elements used within these apps, and (4) determine the reasons for incorporating gamification elements into mobile apps.

**Methods:**

We searched MEDLINE, PsycINFO, CINAHL, Embase, the ACM Digital Library, and IEEE Xplore databases to identify papers published between 2008 and 2023. Original empirical research studies reporting on gamified mobile apps for children with disabilities that implemented at least 1 gamification strategy or tactic were included. Studies investigating serious games or full-fledged games were excluded.

**Results:**

A total of 38 studies reporting on 32 unique gamified mobile apps were included. Findings showed that gamified apps focus on communication skills and oral health in children with autism spectrum disorder while also addressing self-management and academic skills for other disability groups. Gamified mobile apps have demonstrated potential benefits across different populations and conditions; however, there were mixed results regarding their impact. The gamification strategies included fun and playfulness (23/32, 72%), feedback on performance (17/32, 53%), and reinforcement (17/32, 53%) in more than half of apps, whereas social connectivity was used as a gamification strategy in only 4 (12%) mobile apps. There were 2 main reasons for integrating gamification elements into mobile apps described in 16 (42%) studies: increasing user engagement and motivation and enhancing intervention effects.

**Conclusions:**

This scoping review offers researchers a comprehensive review of the gamification elements currently used in mobile apps for the purposes of treatment, education, symptom management, and assessment for children with disabilities. In addition, it indicates that studies on certain disability groups and examinations of health-related outcomes have been neglected, highlighting the need for further investigations in these areas. Furthermore, research is needed to investigate the effectiveness of mobile-based gamification elements on health and health behavior outcomes, as well as the healthy development of children with disabilities.

## Introduction

### Background

Worldwide, approximately 93 million children have a moderate to severe disability, and 13 million children have a severe disability [1]. Children with disabilities face numerous barriers to accessing health services and health-promoting activities [2]. Despite the abundance of research investigating different interventions to improve the lives of children with disabilities, the interventions have not been successfully implemented, limiting the impact of research on public health outcomes [3]. To address this issue, innovative technological advances could significantly improve the health and well-being of marginalized groups [4,5], such as children with disabilities, their families, and the systems of care surrounding them.

Mobile apps, as examples of innovative technological tools, are becoming important in improving access to therapeutic interventions and diagnoses for underserved groups [6]. Mobile health (mHealth), a young but rapidly evolving field, enables the delivery of planned interventions and practices via mobile devices and apps, downloaded and installed on mobile devices to perform a particular task [7]. Constant availability, broader access, fairness of service offerings, personalized content, lower cost, and increased service capacity and efficiency are some advantages of mHealth [8]. Therefore, mHealth can be a tool to create more accessible services for children and adolescents with disabilities and their families as applied to various health-related situations.

There is a growing interest in incorporating game-like elements, called “gamification,” in mobile apps to promote greater engagement with the technology and motivation to achieve specific personalized goals [9,10]. Gamification is the application of various game strategies and tactics in nongame contexts [11-13]. Gamification aims to change individual behavior through a combination of game elements (often delivered within games but also through mobile apps) [14,15] in contrast to “serious games” that is “any form of interactive computer-based game software for one or multiple players to be used on any platform and that has been developed with the intention to be more than entertainment” [16]. Although gamification is a promising concept [14], the empirical research regarding its applications is still in its early stages.

Gamification is increasingly being applied in mHealth to promote healthy behaviors using a wide range of game elements, including challenges, goal setting, feedback, progress bars, points, and levels [9]. There is an increasing trend toward incorporating gamification in different health domains, such as pediatric rehabilitation, physical activity, and chronic health conditions [5,17,18]. Gamification in rehabilitation can enhance therapeutic adherence and can be used to complement traditional interventions for children with disabilities and promote physical activity and other healthy behaviors [19]. The implementation of gamification has the potential to enhance individuals’ adherence to medical protocols and successfully manage their health conditions [20-22]. The inclusion of social support as a gamification component has been recognized as encouraging for enhancing one’s social abilities [23-25]. Previous research has also indicated that the use of gamification has the potential to trigger desirable emotional experiences and enhance users’ levels of satisfaction and self-esteem [26-30]. Furthermore, game design components have become more accessible, cost-effective, and enticing as video games have gained popularity [10].

Ryan et al [31] introduced an integrative process model that incorporates the fundamental elements of self-determination theory, which is a motivational theory. They argued that actions can support or thwart the satisfaction of basic psychological needs, namely autonomy, competence, and relatedness and consequently influence the quality of motivation. Depending on whether the individual’s needs are supported or not, it may then influence mental health outcomes (eg, depression and anxiety) and physical health outcomes (eg, exercise and weight control) [31]. Research has demonstrated that gamification can both facilitate and diminish intrinsic motivation [32]. Therefore, the integration of gamification features in mobile apps entails certain nuances.

Gamification elements such as rewards have the potential to enhance motivation toward continued performance and consequently healthier behaviors; however, numerous research studies indicate that the use of extrinsic motivators or the provision of controlling feedback can significantly diminish intrinsic motivation by impeding individuals’ sense of autonomy [33,34]. The presence of increased levels of extrinsic motivation in the context of gamification is not sufficient as the only criterion for evaluating its advantages [35]. Cheating may also escalate as individuals get involved in attempts to attain greater levels of achievement, primarily driven by the rewards [23] Furthermore, there is a prevailing prediction that a significant proportion of gamification implementations will be doomed to failure because of inadequate understanding regarding the effective design principles of gamification [36]. The development of gamified health solutions frequently lacks collaboration with health professionals, potentially compromising their efficacy and diminishing their credibility [23,28]. When gamification to promote health fails to prioritize the user-centered approach and neglects to consider the unique attributes and demographic factors of potential users, their effectiveness may be undermined [23,28,37,38].

As a result, tailoring the gamification features based on the users’ profiles is crucial to enhance their engagement [39]. Given the diverse needs of children with disabilities, it is imperative for researchers and mobile developers to possess a comprehensive understanding of gamification principles and strategies. This knowledge will enable them to effectively customize gamification features to cater to the specific requirements of this target population. Although there is a growing interest in using gamification elements in mobile apps, there is still a lack of comprehensive understanding in the field of childhood disability. Currently, there is no literature review investigating gamification in mobile apps designed for children with disabilities.

Our scoping review aimed to bridge the following knowledge gaps:

First, there is a deficiency in the systematic identification and categorization of gamified mobile apps (eg, treatment and educational) that are specifically designed for children with disabilities. Understanding the existing evidence in this niche field is crucial to evaluating the scope and diversity of available gamified mobile apps.

Second, there is a lack of comprehensive documentation on the specific health-related outcomes these apps target. Understanding the existing evidence will help us recognize which health-related outcomes have been targeted in this population and help identify disabilities and health-related outcomes that could have been neglected.

Third, there is a lack of comprehensive documentation on the characteristics of gamification strategies and tactics used in these mobile apps. A comprehensive review of gamification types and levels is required to understand how the game elements address the unique needs of children with disabilities.

Fourth, the underlying justification for incorporating gamification elements into mobile apps for this specific group remains unclear.

### Objective

Addressing the abovementioned knowledge gaps is imperative to advance the use of gamification in mobile apps for children with disabilities. Therefore, this review aimed to explore the current use of gamification strategies and tactics in mobile apps for children with disabilities. The four specific objectives are as follows: (1) to identify gamified mobile apps designed for children with disabilities, (2) to identify health-related outcomes that these mobile apps aim to target, (3) to identify the different types and levels of gamification strategies and tactics implemented in these mobile apps (4) to determine the reasons for incorporating gamification elements into mobile apps

## Methods

### Overview

Scoping reviews help identify the types of current literature in a specific field and key characteristics related to a particular context, and analyze the knowledge gaps, while systematic reviews investigate the conflicting results and address any variation in current practices, or compare new interventions against gold standard, established interventions [40]. As there was no current review exploring the current evidence in gamification for children with disabilities and identifying the types of gamification elements in these mobile apps, we sought a scoping review to answer our research objectives. The methodological frameworks proposed by Arksey and O’Malley [41] and the PRISMA-ScR (Preferred Reporting Items for Systematic Reviews and Meta-Analyses extension for Scoping Reviews) checklist [42] (Multimedia Appendix 1) were used to guide this scoping review.

### Search Strategy

The database searches were performed in November 2023 in the following web-based databases: MEDLINE, PsycINFO, CINAHL, Embase, the ACM Digital Library, and IEEE Xplore. The selection of databases, keywords, and relevant indexing (eg, Medical Subject Headings and other database-specific search techniques) were finalized in collaboration with the experienced librarian. The full search strategy is presented in Multimedia Appendix 2. In summary, we had 2 main themes: children with disabilities (population) and gamification in mobile apps (exposure). Regarding the full search strategy used on MEDLINE for the first theme, we combined different key disability terms (lines 1-51) with pediatric population terms (lines 53-55) and parent-related terms (lines 57-62). For the second theme, we combined key terms for mobile apps (lines 64-67) and gamification (line 68). The combination of these 2 themes helped us find any papers that studied mobile apps for children with disabilities. The inclusion criteria for the selection of the papers are discussed in Inclusion Criteria section. To ensure the comprehensiveness of the search, the primary author (EM) manually searched the reference lists of the relevant studies and existing reviews. Furthermore, EM searched the JMIR homepage [43], where no new studies were found. All the research results found in the databases were imported to the Rayyan reference manager website [44], where duplicates were identified and removed.

### Inclusion Criteria

We included a peer-reviewed research article if the conditions presented in Textbox 1 were met.

**Inclusion criteria for included peer-reviewed research articles.**

**Inclusion criteria**

- Publication language: there were no limits imposed on the language of the studies.
- Type of publication: peer-reviewed journal articles and conference proceedings
- Type of study: qualitative, quantitative, or mixed methods
- Time: published between January 2008 and November 2023. The reason for the selected start date was that the App Store and Google Play were launched in 2008, and almost all mobile apps were developed after 2008. Furthermore, another reason for the start date was that the concept of gamification was first introduced by Deterding et al [13] in 2008
- Population: children (aged up to 18 years) with any of the following disabilities: autism spectrum disorder, developmental delays, cerebral palsy, attention-deficit/hyperactivity disorder, dyslexia, intellectual disabilities, Turner syndrome, deglutition disorders, child behavior disorders, speech disorders, sensory disorder, motor disability, brain injuries (eg, traumatic brain injury), or any other brain-based disabilities
- Exposure: mobile apps on any device (smartphone, tablet, or iPad) and platform (Android or iOS) designed for children with disabilities. The mobile apps were included if they incorporated at least 1 gamification element (gamification strategy or tactics)
- Outcome: any health-related outcome that relates to the child’s developmental functioning and general health status

We did not include theses, dissertations, protocols, abstracts, and letters to the editor; however, their references were screened for relevant studies. Nongamified mobile apps were excluded. Furthermore, apps labeled as “serious games” were excluded as they are complete games and fall outside of the scope of this review. Given the unique characteristics of mental health conditions and other disorders such as obesity and cancer in children and adolescents, we excluded these disorders; however, if there was any health-related comorbidity among children with disabilities (eg, if the study was on children with disabilities who are obese), we included them.

### Study Selection

First, we tested the selection criteria, with 2 reviewers (EM and PYY) screening titles and abstracts independently until we reached an interrater agreement of 90%. The same process was followed for the full-text review of potentially relevant studies but with 2 dyads of reviewers (EM and PYY and EM and AC). Upon full-text screening, 1 reviewer (EM) manually searched the target journal and the reference lists of the included articles, abstracts, protocols, etc, and no relevant articles were found. Any disagreements were resolved through discussion to reach a consensus on a final decision, or a third adjudicator was implicated (KS and RC).

### Data Abstraction and Charting

The data extraction form was developed and calibrated among each dyad of reviewers (EM and PYY and EM and AC) with 3 random articles. As the percent agreement was greater than 90% in each dyad, the data abstraction of the remaining articles began, and the conflicts were resolved through discussion. For each study, we extracted data on the study’s first author, country, study design, population (eg, autism), sample characteristics (eg, size and age), mobile app name, device (smartphone, tablet, or iPad), platform (Android or iOS), app purpose, type of gamification strategy and tactics, health-related outcomes, and any reasons for implementing gamification in the mobile apps.

### Data Synthesis and Analysis

Both quantitative and qualitative analyses were performed. A frequency analysis was conducted to illustrate the distribution of studies by publication year, country of origin, disability type in studies, gamification strategies and tactics, and the gamification level incorporated by mobile apps.

The gamification framework proposed by Cugelman [12] was used to assess the gamification elements present in these mobile apps. This framework consists of two sections: (1) gamification *strategies*, which are the persuasive principles of gamification and (2) gamification *tactics*, which are the on-screen features of gamification that app users interact with. The concepts of gamification tactics and strategies proposed by Cugelman [12] were used to operationalize gamification in this review. This framework consists of 7 gamification strategies and 10 gamification tactics.

Descriptive statistics were calculated to examine the level of gamification incorporation into mobile apps, as we wanted to understand if the number of gamification features might influence the outcomes. As there is no previous research exploring the level of mobile-based gamification for children with disabilities, we used arbitrary cutoff points to estimate the gamification level used in previous research in a different field [45]. The level of gamification strategies was labeled as none (no gamification strategies), low (1-2 gamification strategies), medium (3-5 gamification strategies), and high (6-7 gamification strategies). Similarly, the level of gamification tactics was classified as none (no gamification tactics), low (1-3 gamification tactics), medium (4-7 gamification tactics), and high (8-10 gamification tactics).

The primary author (EM) performed the content analysis to identify the health-related outcomes targeted by these mobile apps and the rationale for applying gamification in the apps. Further verification was done through discussion and collaboration with another author (RC) with expertise in conducting reviews and data synthesis.

## Results

### Study Selection

**Figure 1:**
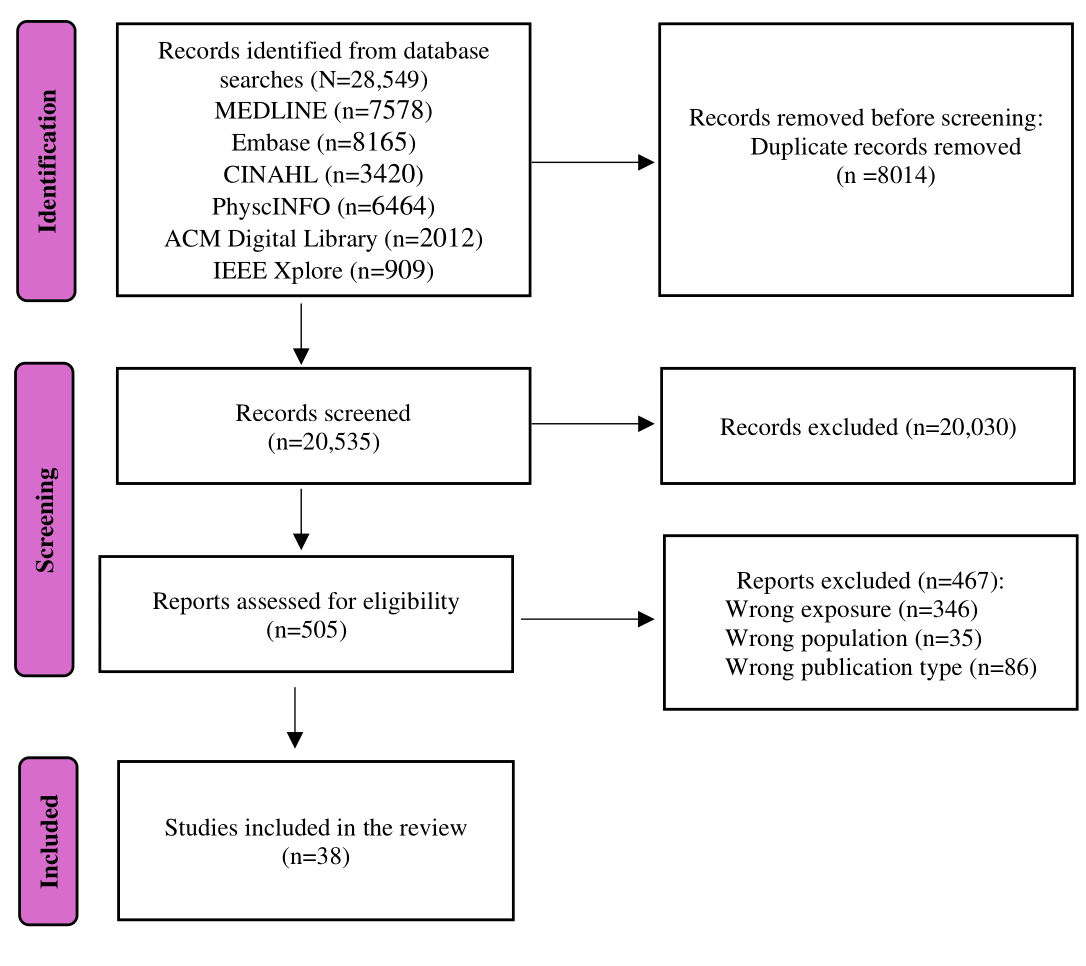
Flowchart of the review process.

The flowchart of the search strategy and study selection is depicted in Figure 1. The initial database search yielded 28,549 citations; after the removal of duplicates, 20,535 (71.93%) citations remained for the title and abstract screening. The first screening phase led to 505 (2.46%) included and 20,030 (97.54%) excluded documents. The studies were excluded because they did not fit our inclusion criteria (eg, wrong population and wrong exposure). The second screening phase consisted of a full-text review of the 505 (2.46%) included documents, resulting in 38 (7.5%) included studies for this scoping review.

### Study Characteristics

Table 1 presents an overview of the study characteristics of all 38 articles included in the scoping review. Although we selected 2008 as the beginning year, all studies were published after 2013, demonstrating that gamification is a recently evolving field. The studies were implemented worldwide, with 37% (14/38) of the studies from Asian countries and 24% (9/38) of the studies from the United States. Most studies (29/38, 76%) used a quantitative research approach and were primarily quasi-experimental (9/29, 31%) or randomized clinical trials (RCTs; 6/29, 21%) studies. All 38 articles included in this review were written in English. Autism spectrum disorder (ASD) was the most common condition reported in 18 (47%) of the 38 included studies, followed by vision impairment (4/38, 11%) and dyslexia (4/38, 11%). Multimedia Appendix 3 [46-83] demonstrates details regarding the study and participant characteristics.

**Table 1:** Characteristics of included studies (n=38).

Characteristics		Value, n (%)
**Year of publication**		
	2018-2023	23 (61)
	2013-2017	15 (39)
	2008-2012	0 (0)
**Country**		
	United States	9 (24)
	Australia	5 (13)
	Malaysia	4 (11)
	United Kingdom	3 (8)
	Canada	2 (5)
	Hungary	2 (5)
	Singapore	2 (5)
	Indonesia	2 (5)
	Spain	1 (3)
	Japan	1 (3)
	Türkiye	1 (3)
	Saudi Arabia	1 (3)
	United Arab Emirates	1 (3)
	India	1 (3)
	Myanmar	1 (3)
	South Africa	1 (3)
	Romania	1 (3)
**Study design**		
	Quantitative	26 (68)
	Mixed methods	8 (21)
	Qualitative	4 (11)
**Disability**		
	Autism spectrum disorder	18 (47)
	Vision impairments	4 (11)
	Dyslexia	4 (11)
	Attention-deficit/hyperactivity disorder	2 (5)
	Mild traumatic brain injury	2 (5)
	Neurodevelopmental disabilities	2 (5)
	Physical disabilities	1 (3)
	Pervasive developmental disorder	1 (3)
	Mild intellectual disabilities	1 (3)
	Complex needs (physical disabilities, learning, and communication difficulties)	1 (3)
	Hearing impairments	1 (3)
	Concussion	1 (3)

### Mobile App Characteristics

Table 2 [46-83] demonstrates results regarding the general characteristics of mobile apps and gamification. A total of 32 unique gamified mobile apps were identified in the 38 included studies. Approximately 44% (14/32) of the identified mobile apps fell into the treatment category, designed to help children with disabilities improve their skill competencies, such as story creation and story sharing, and social communication. Educational (n=13, 41%) and assessment (n=3, 9%) apps were the second and third most observed categories. One app was specifically designed for managing symptoms.

**Table 2:** Summary descriptions of studies included in the scoping review.

Study, year	App name	App category	Health-related outcome	Gamification strategies	Gamification tactics
Kucirkova et al [46], 2014	Our story	Treatment	Social communication and story-telling abilities	Goal setting, capacity to overcome challenges, fun, and playfulness	Provides clear goals and offers a challenge
Moore et al [47], 2015	TOBY	Treatment	Different rehabilitation goals, such as sensory awareness, imitation, and social interaction	Goal setting, feedback on performance, reinforcement, comparing progress, fun, and playfulness	Offers a challenge, levels, points, shows progress, feedback, and gives reward
Parsons et al [48], 2020	TOBY	Treatment	Different rehabilitation goals, such as receptive language, social skills, and pragmatic language	Goal setting, feedback on performance, reinforcement, comparing progress, fun, and playfulness	Offers a challenge, levels, points, shows progress, feedback, gives reward
Parsons et al [49], 2019	TOBY	Treatment	Social communication	Goal setting, feedback on performance, reinforcement, comparing progress, fun, and playfulness	Offers a challenge, levels, points, shows progress, feedback, and gives reward
Penev et al [50], 2021	Guess what	Treatment	Social communication	Goal setting, the capacity to overcome challenges, feedback on performance, reinforcement, fun, and playfulness	Provides clear goals, offers a challenge, points, feedback, gives rewards, and provides badges
Saputra [51], 2016	LexiPal	Educational	Enjoyment and motivation in learning	Goal setting, feedback on performance, reinforcement, fun, and playfulness	Provides clear goals, levels, points, feedback, gives rewards, provides badges, and story or theme
Schmidt et al [52], 2020	SMART	Treatment	Self-management and relaxation	Goal setting, feedback on performance, reinforcement, fun, and playfulness	Provides clear goals, feedback, gives rewards, provides badges, and story or theme
Thida et al [53], 2020	VOIS	Treatment	Language	The capacity to overcome challenge, feedback on performance, reinforcement, fun, and playfulness	Offers a challenge, shows progress, feedback, and give rewards
Urakami [54], 2021	GROWJECTOR	Treatment	Medication adherence	Goal setting, reinforcement, and social connectivity	Provides clear goals, points, and gives rewards
Ying et al [55], 2016	NR^a^	Educational	Learning road safety	Fun and playfulness	Story or theme
Chua et al [56], 2017	NR	Educational	Emotional learning	Feedback on performance, fun, and playfulness	Feedback and story or theme
Doenyas et al [57], 2014	NR	Educational	Sequencing skill	Goal setting, the capacity to overcome challenges, feedback on performance, and reinforcement	Offers a challenge, feedback, and points
Holmes et al [58], 2016	NR	Treatment	Visual acuity	Reinforcement and compare progress	Levels, points, and shows progress
Kelly et al [59], 2016	Dig Rush	Treatment	Visual acuity	Goal setting, fun, and playfulness	Levels and points
Aburukba et al [60], 2017	AutiAid	Treatment or symptoms management	Memory and management of symptoms	Goal setting and social connectivity	Provides clear goals, levels, and points
Alnaghaimshi et al [61], 2020	AutismWorld	Assessment and educational	User’s literacy on autism	Social connectivity	—^b^
Barta et al [62], 2017	AutiSoft	Symptoms management	Manage daily routines	Feedback on performance and reinforcement	Feedback and gives rewards
Birtwell et al [63], 2019	SideKicks!	Treatment	Social communication	Goal setting, fun, and playfulness	Provides clear goals and story or theme
Borhan et al [64], 2018	Mr. Read	Educational	Reading skills	Feedback on performance, reinforcement, fun, and playfulness	Points, feedback, shows progress, and story or theme
Brkic et al [65], 2022	FarmApp	Assessment	No specific behavior	Feedback on performance, reinforcement, fun, and playfulness	Offers a challenge, feedback, points, and story or theme
Daud and Abas [66], 2013	Dyslexia Baca	Educational	Letter recognition	Feedback on performance, reinforcement, fun, and playfulness	Feedback, gives reward, and story or theme
Dehkordi and Rias [67], 2014	GO-Go	Educational	Multiple cues responding	Reinforcement, fun, and playfulness	Offers a challenge, gives rewards, and story or theme
Gómez and Carro [68], 2014	AdaptADHD	Treatment	Concentration and impulse control abilities	Goal setting	Provides clear goals and levels
Guzsvinecz et al [69], 2017	Sliders	Educational	Logical thinking and deductive reasoning	Feedback on performance and reinforcement	Points, feedback, and shows game leaders
Hu et al [70], 2019	NeuroCare	Symptoms management	Self-management of pediatric concussion	Goal setting and feedback on performance	Provides clear goals and feedback
Irwin et al [71], 2015	Listening to Faces (L2F)	Educational	Audiovisual speech perception	Feedback on performance, compare progress, fun, and playfulness	Feedback and shows progress
Kalantarian et al [72], 2019	Guess what	Treatment	Social communication	Goal setting, the capacity to overcome challenges, feedback on performance, reinforcement, fun, and playfulness	Provides clear goals, offers a challenge, points, feedback, gives rewards, and provides badges
Macdonald et al [73], 2022	NR	Educational	Reading skills	Feedback on performance, reinforcement, fun, and playfulness	Feedback, gives rewards, and story or theme
Manh et al [74], 2018	NR	Treatment	Visual acuity	Capacity to overcome challenges and reinforcement	Levels and points
Mwamba et al [75], 2019	Paediatric Attention-Deficit/Hyperactivity Disorder Application Software (PANDAS)	Assessment	No specific behavior	Capacity to overcome challenges, reinforcement, fun, and playfulness	Offers a challenge, points, and story or theme
Tang et al [76], 2021	ColourSpot	Assessment	No specific behavior	Feedback on performance, reinforcement, Fun and playfulness	Feedback, gives rewards, and story or theme
Cahyono [77], 2022	LexiPal	Educational	Enjoyment and motivation in learning	Goal setting, feedback on performance, reinforcement, fun, and playfulness	Provides clear goals, levels, points, feedback, gives rewards, provides badges, and story or theme
Chistol et al [78], 2023	Autism Assistant	Treatment	Multiple behavioral skills	Fun and playfulness, goal setting, compare progress, and capacity to overcome challenges	Story or theme, provides clear goals, shows progress, and offers a challenge
Tan et al [79], 2023	NUS care	Educational	Oral health	Fun and playfulness, reinforcement, and social connectivity	Story or theme, points, and rewards
Schmidt et al [80], 2022	Self-Monitoring Activity Regulation and Relaxation Treatment (SMART)	Treatment	Self-management and relaxation	Goal setting, feedback on performance, reinforcement, fun, and playfulness	Provides clear goals, feedback, gives rewards, provides badges, and story or theme
Johnson et al [81], 2022	Zingo	Treatment	Therapy adherence	Fun and playfulness, feedback on performance, reinforcement, goal setting, and compares progress	Feedback, points, story or theme, gives rewards, provides clear goals, and shows progress
Johnson et al [82], 2023	Zingo	Treatment	Therapy adherence	Fun and playfulness, feedback on performance, reinforcement, goal setting, and compares progress	Feedback, points, story or theme, gives rewards, provides clear goals, and shows progress
Krishnan et al [83], 2021	Brush Up	Educational	Oral health	Fun and playfulness, reinforcement, feedback on performance	Story or theme, feedback, and gives rewards

^a^NR: not reported.

^b^Not applicable.

A variety of health-related outcomes were identified, including a wide range of developmental, therapeutic, and educational skill competencies. Social communication (5/32, 16%), self-management (4/32, 11%), visual acuity (3/32, 9%), and oral health (2/32, 6%) were the most observed outcomes in the review. Only 2 (6%) studies investigating the LexiPal app targeted psychological outcomes such as motivation and engagement.

Multimedia Appendix 3 demonstrates other details regarding the participant and mobile app characteristics [46-83]. Regarding the 32 platforms, 14 (44%) apps were exclusively designed for iOS and 10 (31%) apps for Android, with 3 (9%) apps available on both platforms. In addition, 3 (9%) studies did not report the platform used. Of the 32 apps, 12 (38%) gamified apps were delivered on smartphones, 10 (31%) exclusively on iPads, 5 (16%) on both smartphones and tablets, 3 (9%) on both smartphones and iPads, and 2 (6%) exclusively on tablets. Most studies (28/32, 88%) did not report on costs related to app development.

### Gamification Characteristics

Table 3 outlines the number and percentage of each gamification strategy and tactic adopted by 32 gamified mobile apps in this review. The most popular gamification strategy among 32 mobile apps was fun and playfulness (n=23, 72%), resulting in a higher number of gamification tactics (on-screen features) such as story or theme, avatars, a graphic representation of story characters, fun videos, and sound effects.

**Table 3:** Number of gamification strategies and tactics (n=32).

			Value, n (%)		Reference
**Gamification strategies**					
	Fun and playfulness	23 (72)		[46-53,55,56,59,63-67,71,73,75,76,78,79,81,82]	
	Feedback on performance	17 (53)		[47-53,56,57,62,64-66,69-73,76,81,82,83]	
	Reinforcement	17 (53)		[47-54,57,58,62,64-67,69,72-77,79-82,83]	
	Goal setting	14 (44)		[46-52,54,57,59,60,63,68,70,72,78,81,82]	
	The capacity to overcome challenges	7 (22)		[46,50,53,57,72,74,75,78]	
	Compares progress	5 (16)		[47-49,58,71,78,81,82]	
	Social connectivity	4 (12)		[54,60,61,79]	
**Gamification tactics**					
	Feedback	18 (56)		[47-53,56,57,62,64-66,69-73,76,81,82,83]	
	Points	14 (44)		[47-51,54,57-60,64,65,69,74,79,81,82]	
	Story or theme	14 (44)		[51,52,55,56,63-67,73,75,76,78,79,81,82,83]	
	Gives rewards	13 (41)		[47-54,62,66,67,72,73,76,79,81,82,83]	
	Provides clear goals	11 (34)		[46,50-52,54,60,63,68,70,72,78,81,82]	
	Offers a challenge	8 (25)		[46-50,53,57,65,67,72,75,78]	
	Levels	7 (22)		[47-49,51,58-60,68,74]	
	Shows progress	7 (22)		[47-49,53,58,64,71,78,81,82]	
	Provides badges for achievements	3 (9)		[50-52,72]	
	Shows game leaders	0 (0)		—^a^	

^a^Not applicable.

Furthermore, more than half (17/32, 53%) of mobile apps adopted feedback on performance and reinforcement (17/32, 53%). This finding is consistent with the high presence of on-screen features such as visual and verbal feedback, providing points and stars, and giving monetary and nonmonetary rewards upon completing a specified task. Finally, social connectivity was the least common gamification strategy observed in the apps (4/32, 12%), resulting from a low presence of on-screen social connectivity features. Only 12% (4/32) apps provided a social connection like a chat room for users where they can send messages [54,60,61,79]. The most common tactics, meanwhile, were feedback, points, and rewards. None of the apps displayed who the game leaders were.

Table 4 demonstrates the levels of gamification strategies and tactics adopted by 32 gamified mobile apps. Only 1 (3%) app did not adopt any gamification tactics [61]. Although more than half (18/32, 56%) of the mobile apps had adopted a medium level of gamification strategies, only 25% (8/32) of the mobile apps had incorporated a medium level of gamification tactics, known as on-screen features.

**Table 4:** Level of gamification incorporated in mobile apps (N=32).

		Value, n (%)	Reference
**Number of gamification strategies adopted**			
	0 (none)	0 (0)	—^a^
	1-2 (low)	13 (41)	[55,56,58-63,67-70,74]
	3-5 (medium)	18 (56)	[46-49,51-54,57,64-66,71-73,75,76,78,79,81,82,83]
	6-7 (high)	1 (3)	[50,72]
**Number of gamification tactics adopted**			
	0 (none)	1 (3)	[61]
	1-3 (low)	23 (72)	[46,54-60,62,63,65-71,73-76,79,83]
	4-7 (medium)	8 (25)	[47-53,64,72,78,81,82]
	8-10 (high)	0 (0)	—

^a^Not applicable.

We identified the rationale for using gamification in apps in 16 (42%) of the 38 included studies. Multimedia Appendix 4 [50-52,56,57,60,62-66,76,77,80-82] presents the complete results. The two most cited reasons were (1) to promote user engagement and motivation and (2) to increase the intervention effects. Some of the underlying reasons for the first theme are as follows: encourage use [52,65] and increase engagement with the intervention [50,81]. For example, the SMART app and FarmApp provided feedback on performance and offered different reinforcement features to keep users more involved with the app content [52,65].

Regarding the second theme, the researchers applied gamification to enhance learning [51,56,63,64] and increase intervention efficacy [66,76,81,82]. As an example, the Zingo app incorporated a digital pet where users, by adhering to the prescribed therapies in the app, receive stars to make changes to avatars. In addition, children and clinicians can monitor their weekly progress in the app.

## Discussion

### Principal Findings

This scoping review aimed to offer an overview of existing research using gamification in mobile apps for children with disabilities. A total of 38 studies and 32 unique mobile apps were identified, and most incorporated a limited number of gamification strategies and tactics. Of the 32 apps, 18 (56%) were specifically designed for children with ASD, while 14 (44%) were for children with other types of disabilities.

Social communication impairments are a clinical indicator of ASD [84]. Notably, mobile apps designed for children with ASD identified in this scoping review were predominantly focused on enhancing communication and social skills [46,49,63,72]. Our review found that 2 educational apps were designed specifically for children with ASD to acquire knowledge and skills related to oral health [79,83].

This review identified 4 (12%) of the 32 mobile apps that specifically target the self-management of children with disabilities. Previous research indicates that effective self-management behaviors could enhance health-related outcomes in children with complex needs [85]. Self-management interventions for individuals with intellectual disabilities primarily target self-management in the workplace, self-management of medical conditions, and self-management of daily activities [86]. Similar to the previous literature, the 4 (12%) mobile apps identified in this review focused on the management of symptoms in children with ASD [60], traumatic brain injury [52,80], and concussion [70] and on managing the daily routines of children with ASD [62].

Children with developmental delays, including those with learning disabilities, ASD, and attention-deficit/hyperactivity disorder, generally have lower academic achievements than those without developmental delays. The most impacted domains are cognitive, attention and memory, visual-motor skills, and behavioral functioning [87]. Similarly, several mobile apps identified in this review targeted memory [60], reading skills [73], letter recognition [66], multiple cues for responding [67], concentration and impulse control abilities [68], audiovisual speech perception [71], and behavioral skills [78] to help children with disabilities attain a higher level of academic achievement.

The major objective of the 9% (3/32) assessment mobile apps in this review was to assess users’ cognitive control and memory [65], visual acuity [76], and screen children with attention-deficit/hyperactivity disorder [75]. These apps did not focus on any specific changes in behavioral, developmental, or other health-related outcomes.

### The Gamified Apps and Their Impact

The findings of our scoping review shed light on the relationship between gamified mobile apps and health-related outcomes in children with different disabilities. Multimedia Appendix 5 [46,48-50,54,57-59,64,72-74,77,83] provides a comprehensive overview of 15 studies that have reported the impact of gamified mobile apps as interventions across different populations and conditions. Quantitative studies show mixed results, with significant improvements in targeted health-related outcomes such as social responsiveness, language skills, and visual acuity. However, studies with comparison groups often reveal that traditional methods (eg, patching for amblyopia) may still be more effective in some cases. For example, in 3 studies on children with amblyopia (a type of visual disorder where usually one eye gets poor vision), the 3 mobile apps had treatment goals that aimed to improve the visual acuity of these children. The results of 2 RCT studies showed an improvement in visual acuity using the mobile app [58,59], whereas in 1 RCT, there was no difference in visual acuity level using the mobile app compared to traditional intervention (patching) and even was less effective [74].

Furthermore, gamified systems can intrinsically motivate individuals to start and maintain the execution of healthy behaviors [88]. A meta-analysis by Bai et al [89] showed that gamification can improve student learning outcomes by fostering motivation among learners. In addition, 2 studies using the LexiPal mobile app among children with dyslexia focused on psychological outcomes such as motivation and engagement [51,77]. The study by Cahyono [77] showed that LexiPal has the potential to increase extrinsic motivation through a reward system and intrinsic motivation through activity levels and fun features; however, a longer intervention is necessary to assess the impact of gamification on long-term motivation and engagement of learners.

Moreover, the importance of user preferences and the need for more personalized gamification is highlighted. For example, the variability in the effectiveness of mobile apps in children with different levels of disability in 2 (5%) of the 38 identified studies suggests that personalization is crucial [46,49]. Personalized (or adaptive) gamification is a method for enhancing the design of game-based systems by tailoring tasks, game rules, and features to match each user’s preferences or skill level [90]. Personalized gamification can be implemented through (1) customization, where users can select the elements they wish to use, and (2) automatic adaptation, in which the system selects the game design elements for each user, potentially with some user input. Therefore, developers and researchers are encouraged to consider integrating personalized gamification elements into their apps to improve user engagement and, consequently, the effectiveness of mobile apps on health-related outcomes.

Finally, it is noteworthy that most (11/15, 73%) identified studies with an intervention did not have comparison groups, which makes it challenging to draw definitive conclusions regarding the effectiveness of various mobile apps.

### Gamification Strategies and Tactics

Following the gamification framework proposed by Cugelman [12], the most common gamification strategies were fun and playfulness, feedback on performance, reinforcement, and goal setting, whereas social connectivity was the least commonly used strategy, followed by comparing progress. Moreover, feedback, points, story or theme, and rewards were the most common on-screen features, while showing game leaders (leaderboards), badges, and showing progress were the least common elements applied to mobile apps.

The fun and playfulness strategy (23/32, 72%) was the gamification principle most applied to mobile apps. There was frequent use of on-screen tactics such as stories, themes, avatars, graphic representations of information, fun videos, and audio effects. Incorporating these features requires a significant amount of computational resources, time, and knowledge [91]. Most (23/32, 72%) of the mobile apps in this review had comparable playfulness elements to enhance children’s experience of fun and motivate them to use the app on a regular basis. Previous studies showed that playful and fun experiences in mobile apps will increase positive attitudes toward mobile apps when the users can engage in pleasurable experiences [92,93].

Feedback on performance and reinforcement were among the most frequently used gamification strategies in mobile apps. This finding aligns with the results of previous research identifying successful behavior-change techniques in gamified mobile apps [56]. Previous reviews on gamification in other populations found that 94% and 81% of health apps had incorporated feedback on performance and reinforcement, respectively, and achievement- and progress-oriented elements such as in-app rewards [12,94]. In our scoping review, the most prevalent types of reinforcement were points (14/32, 44%) and tangible rewards (13/32, 41%), aligning with previous reviews, and indicating a positive direction toward promoting health behavior change through these strategies. This finding also emphasizes that easy-to-implement game features, such as points and feedback through messaging are the most widely used gamification features to promote engagement and motivation [94]. Nevertheless, the outcomes were frequently measured only through in-app behavior (eg, completing tasks in the app for rewards).

We found that achievement- and progress-oriented rewards were given to users as a result of their change in specific behaviors, such as completing cognitive assessments [65] or participating in daily language test challenges [66]. For instance, when children with ASD used the TOBY iPad app, an early intervention tool, they had to choose a specific picture from a set of pictures. Upon completing the task, they would gain tokens (points), which could be used to choose a reward [47-49]. Another app, LexiPal, an educational app for children with dyslexia, used various game elements, such as points, feedback, and rewards. Upon successfully completing 1 round of tasks, a pop-up window would appear to illustrate the score and reward. If the child gets a score of 4 to 5, they earn a golden cup reward and receive text and audio feedback [51]. Users were rarely rewarded for behavior changes external to the app. For instance, in the Urakami app, users who completed outpatient therapy sessions could collect points, which could be exchanged to purchase in-app avatars [54].

A growing body of literature has criticized the paucity of use of incentives through points, badges, and leaderboard elements in digital health solutions [95]. Points were among the most frequently applied game elements in our review, whereas badges and leaderboards were incorporated by a minority of apps. Many studies have investigated the effectiveness of a combination of points, leaderboards, and badges to increase outcomes such as engagement in physical activity. Several studies have shown that the application of these game features could significantly enhance individuals’ physical activity [20]. In contrast, several studies found that points and leaderboards did not significantly impact walking compared to a nongamified version of the same app [96,97]. In addition, Maher et al [98] found that a combination of rewards and leaderboards led to a short-term increase in physical activity but there was no long-term positive impact on health behavior. Further research is needed to investigate the impact of using game features for short- and long-term impacts in the childhood disability field.

Goal setting is a known intervention strategy for successful health behavior change [99]. In our review, 44% (14/32) of the mobile apps used goal setting to promote user engagement. Previous research has outlined that combining goal setting with showing progress, feedback, and rewards can significantly enhance intrinsic motivation toward behavior [100]. Although feedback and rewards have been extensively applied to the identified apps in our review, comparing progress elements has been underused. There is a vast amount of literature on the possible benefits of rewards and feedback, yet each element’s effectiveness still needs to be determined [101]. Another concern is that these features may enhance extrinsic motivation rather than intrinsic motivation, which leads to the weak maintenance impact of gamified apps [102]. Therefore, we recommend further investigating the independent effects of individual mobile-based gamification elements on children with disabilities.

Despite the potential advantages of social connectivity on young people’s well-being [103], only 4 (12%) of the 32 apps implemented a social connectivity strategy. They provided the users with access to chat rooms [61], the ability to share the points with parents [60], and the ability to send messages to parents and health professionals [54,79]. The scarcity of social connectivity options found in our review contrasts with previous research indicating that social networks could positively impact health behavior change insofar as app users can interact with other users and share their points and experiences with one another. [103,104]. A recent review identified several social support features where app users could interact with others through sharing posts and sending private messages [105]. However, previous studies highlighted the potential negative aspects of social connectivity in mobile apps. For example, concerns were raised about inappropriate content sharing and messaging between children and information inaccuracy in the technology space [106,107]. Therefore, while much research on the potential effectiveness of social connectivity has been carried out, some critical issues need to be investigated in the childhood disability field. Moreover, no apps provide leaderboards. This may suggest a deliberate decision in the design, as children with disabilities are generally a particularly susceptible group who may experience increased levels of stress when comparing themselves to their peers [108,109]. A meta-analysis of qualitative studies of students shows that gamification can cause anxiety and jealousy among students [89]. For example, Johnson et al [81] did not incorporate some traditional gamification elements, such as badges and leaderboards, considering the needs of children with neurodevelopmental disabilities in their study. Therefore, researchers in this field should investigate the specific needs and potential stressors of children with disabilities when considering the incorporation of gamification elements such as leaderboards.

### Level of Gamification and Reasons to Apply Gamification

This review highlights that most (18/32, 56%) identified apps implement a medium level of gamification strategies and a low level of gamification tactics, with few (1/32, 3%) adopting a high level of gamification strategies or tactics. The Guess What app used the greatest number of gamification tactics (6 of 7). All identified apps in our review used at least 1 gamification strategy, which supports previous research findings that gamification is meant to significantly improve psychological outcomes [110,111]. While gamification tactics, also known as on-screen features, are considered to be part of persuasive app design to promote engagement and motivation [12], most identified apps implemented a low (23/32, 72%) number of gamification tactics. Children with disabilities are an underserved group that faces numerous barriers to accessing health services [112]. Designing digital solutions for children with disabilities requires collaboration among childhood disability researchers, mHealth experts, and children and their families. The provision of these solutions such as gamified mobile apps for children with disabilities has the potential to reduce health inequities.

However, the level of incorporated gamification tactics should be interpreted with caution in our review. One vital theoretical issue is that multiple gamification frameworks have different definitions of gamification and categorizations of game elements. For example, Lister et al [11] used “gamification” to define levels, rewards, prizes, and competitions but not avatars; meanwhile, Johnson et al [110] used “gamification” to describe all these game elements. Similarly, multiple studies separate feedback and rewards [11,113], whereas Sardi et al [9] counted them as 1 game mechanic. Although we used the framework proposed by Cugelman [12] to define gamification in our study, it would be difficult to make a definite conclusion regarding the level of gamification, as the number of game elements varies in different frameworks. Therefore, there is a need to have a solid framework for mobile-based gamification for childhood disabilities.

Furthermore, researchers applied gamification to apps for various purposes. We found justification for applying particular game elements in 16 studies. Of the 16 studies, 9 (56%) used gamification elements to promote engagement and motivation. Gamification aims to include playful elements to transform a typically boring activity into one that is enjoyable and engaging [9]. For example, FarmApp, which is a mobile app used to assess cognitive skills among children with neurodevelopmental disabilities, incorporated interactive game-like elements to be more motivating and enjoyable for children to complete the assessments [65]. In addition, the SMART app, which is for the self-management of children with traumatic brain injury, was redesigned and implemented gamified components to encourage youth with mild traumatic brain injury to use the app daily and manage their symptoms [52]. These findings align with the purpose of gamification as a tool to increase engagement and motivation [10,12].

In contrast, gamification was also applied to increase the impact of the intervention. Many mobile apps included game elements to increase the efficacy of the intervention. For instance, gamification was used in the ColourSpot app to encourage users to complete the intervention [76]. In the Dyslexia Baca app, visual graphics were incorporated to assist children with dyslexia in understanding the intervention instructions [66]. In another example, Johnson et al [81] used various gamification tactics, such as avatars, weekly progress monitoring, and earning stars, to engage children in their therapy prescription app. These justifications align with the previous literature showing that engaging apps, such as gamified apps, can enhance the effectiveness of interventions by encouraging users to use them consistently and frequently [114]. Although the capacity of gamification to promote engagement and motivation has been extensively studied [10,12,110], more research is needed to confirm the ability of gamification to increase intervention efficacy.

### Limitations and Recommendations

Although this scoping review was guided by the PRISMA-ScR framework [42], it has some limitations. First, the primary aim of our review was to summarize a record of all gamified apps for children with disabilities from 2008 to 2023; however, we did not assemble any information on the effectiveness of gamified apps on any evaluation metric. By including broad search keywords in the search strategy, we had a high volume of document titles and abstracts to screen. Nevertheless, this enhances the risk of accidental exclusion of relevant citations. To minimize this, before both abstract and full-text screening, we performed pilot testing on a random sample of documents, and any discrepancies were resolved by KS. This ensured that the title and abstract screening was appropriate before the full-text screening.

In addition, although our scoping review was inclusive (no restrictions on study design), we excluded studies of children with mental concerns (eg, anxiety and depression) or other health issues (eg, obesity and cancer). Given the unique characteristics of mental health problems in children and adolescents, we recommend an independent review of gamified mobile apps for children with mental health issues.

Furthermore, unpublished studies were not included in this scoping review. Because many mHealth apps are privately designed, development or evaluative information for these apps is not available in the public domain, which may result in a substantial knowledge gap. Although private companies have been increasingly transparent in publishing data in recent years [115], this knowledge gap cannot be addressed in this scoping review. Therefore, the results of this review are not generalizable to commercial apps for children with disabilities.

Although the consultation exercise is a vital yet optional component of the scoping review framework proposed by Arksey and O’Malley [41], it was not conducted in this review. Specifically, we are conducting a separate project to seek out stakeholder input to further inform this area of research. The primary author (EM) used the results of this scoping review to inform the interview guide of a qualitative project where different stakeholders, including children and youth with disabilities, parents or caregivers, clinicians, and representatives of community organizations, shared their perspectives about different gamification elements. The findings from this research will not only enhance the results of our scoping review but also make an important contribution to the deeper understanding of best practices in developing gamified mobile apps for children with disabilities.

Scoping reviews aim to rigorously survey the current body of literature and identify crucial concepts, types of evidence, and knowledge gaps. Typically, they are not structured to evaluate the effectiveness of the interventions. In accordance with this methodology, our review did not assess the effectiveness of the identified gamified mobile apps; however, we reported a summary of the mobile app’s impact on child outcomes. Indeed, systemically evaluating the effectiveness would be challenging, given the considerable heterogeneity in the types of disabilities, mHealth strategies used, and the wide range of outcomes applied. Recognizing this limitation, there is a need for future research to evaluate the effectiveness of these gamified mobile apps on specific populations and outcomes.

Finally, limited evidence was provided in this review on the extent to which health behaviors “outside the app” were augmented in children with disabilities, and its association with the gamification features proposed. Johnson et al [110] conducted a review to assess the impact of gamified interventions on health and well-being in a broader population indicating that gamification could have a positive impact on healthy behaviors (eg, physical activity). Future research should investigate the association of gamification features in mobile apps with subsequent healthy behaviors “outside the app” for children with disabilities.

### Conclusions

This review provides a summary of the current literature on mobile-based gamification used for children with disabilities reported after 2008. A total of 6 databases were comprehensively searched, and 38 studies with 32 unique apps were identified that focused predominantly on treatment goals and were in most cases used in children with ASD. This review demonstrates that gamified mobile apps for children with ASD are mainly designed to enhance communication, social skills, and oral health knowledge. In addition, several mobile apps address self-management in various conditions, academic achievements in learning disabilities, and psychological outcomes such as motivation and engagement, demonstrating their potential in improving diverse health-related outcomes in children with disabilities. The results of this study showed that gamification could provide potential benefits across different populations and conditions; however, there were mixed results regarding its impact and benefits. These results can guide other researchers in the childhood disability field in recognizing disabilities or behavioral outcomes that have been neglected, thus informing future mobile app development and research on those disabilities. Collectively, this information will enable the researchers in this field to understand how gamification can improve intervention effects on relevant outcomes and meet the specific needs of this population.

## Appendix

Multimedia Appendix 1 PRISMA-ScR (Preferred Reporting Items for Systematic Reviews and Meta-Analyses extension for Scoping Reviews) checklist.

Multimedia Appendix 2 Search strategy.

Multimedia Appendix 3 Summary descriptions of studies included in the scoping review.

Multimedia Appendix 4 Reasons for applying gamification found in scoping review.

Multimedia Appendix 5 Included studies with results directly related to an intervention (n=15).

## Abbreviations

ASD: autism spectrum disorder
mHealth: mobile health
PRISMA-ScR: Preferred Reporting Items for Systematic Reviews and Meta-Analyses extension for Scoping Reviews
RCT: randomized clinical trial

## Data Availability

All data generated or analyzed during this study are included in this published article and its supplementary information files.
