# Supplementary material for: Gamification in Mobile Apps for Children With Disabilities: Scoping Review
Source: JMIR Serious Games. 2024 Sep 6;12:e49029. doi: 10.2196/49029 (PMC11415723; doi:10.2196/49029)
Supplement: Multimedia Appendix 2 [file games_v12i1e49029_app2.docx]

**Medline**

1 exp Developmental Disabilities/ or Developmental Disabilit*.mp.

2 Fragile X Syndrome.mp. or exp Fragile X Syndrome/

3 exp Learning Disabilities/ or Learning Disabilit*.mp.

4 dyscalculia.mp. or exp Dyscalculia/

5 dyslexia.mp. or exp Dyslexia, Acquired/ or exp Dyslexia/

6 dyslexic.mp.

7 neurodevelopmental disabilit*.mp.

8 exp Neurodevelopmental Disorders/ or neurodevelopmental disorder*.mp.

9 exp Intellectual Disability/ or Intellectual Disabilit*.mp.

10 Motor Skills Disorder*.mp.

11 Stereotypic Movement Disorder*.mp.

12 Reactive Attachment Disorder*.mp.

13 exp Child Development Disorders, Pervasive/ or Child Development Disorder*.mp.

14 Child Behavior Disorders/ or Child Behavior Disorder*.mp.

15 (Attention Deficit and Disruptive Behavior Disorder*).mp.

16 (autism or Asperger*).mp.

17 exp Autistic Disorder/ or autistic.mp.

18 (developmental delay or developmental coordination disorder).mp.

19 Cerebral Palsy.mp. or exp Cerebral Palsy/

20 exp Attention Deficit Disorder with Hyperactivity/ or attention deficit disorder*.mp.

21 Attention Deficit Disorder with Hyperactivity.mp.

22 ADHD.mp.

23 attention deficit hyperactive disorder*.mp.

24 exp Brain Injuries, Traumatic/ or exp Brain Injuries/ or brain injur*.mp.

25 Shaken Baby Syndrome.mp.

26 Traumatic Brain Injur*.mp.

27 Turner Syndrome.mp.

28 exp Mental Disorders/ or mental disorder*.mp.

29 exp Deglutition Disorders/ or Deglutition Disorder*.mp.

30 exp Child Behavior Disorders/ or Child Behavior Disorder*.mp.

31 exp Speech Disorders/ or Speech Disorder*.mp.

32 Aphasia.mp.

33 Articulation Disorder*.mp.

34 Echolalia.mp.

35 Stuttering.mp.

36 Mutism.mp.

37 motor disabilit*.mp.

38 motor disorder*.mp.

39 traumatic brain injur*.mp.

40 sensory integration.mp.

41 Down Syndrome.mp. or exp Down Syndrome/

42 Mental Retardation.mp.

43 Williams Syndrome.mp. or Williams Syndrome/

44 exp Sensation Disorders/ or sensory disorder*.mp.

45 Hearing Disorder*.mp.

46 hearing loss.mp.

47 Olfaction Disorder*.mp.

48 Somatosensory Disorder*.mp.

49 Taste Disorder*.mp.

50 Vision Disorder*.mp.

51 blindness.mp.

52 1 or 2 or 3 or 4 or 5 or 6 or 7 or 8 or 9 or 10 or 11 or 12 or 13 or 14 or 15 or 16 or 17 or 18 or 19 or 20 or 21 or 22 or 23 or 24 or 25 or 26 or 27 or 28 or 29 or 30 or 31 or 32 or 33 or 34 or 35 or 36 or 37 or 38 or 39 or 40 or 41 or 42 or 43 or 44 or 45 or 46 or 47 or 48 or 49 or 50 or 51

53 exp infant/ or exp child/ or adolescent/ or exp pediatrics/

54 (child* or pediatric* or paediatric* or prematur* or preterm* or perinat* or neonat* or neo nat* or newborn* or new born* or infan* or baby* or babies or toddler* or boy* or girl* or kid$1 or school* or juvenil* or underage* or under age* or teen* or minor$1 or youth$1 or adolescen* or pubescen* or puberty).mp.

55 (neonat* or infan* or child* or adolescen* or pediatric* or paediatric*).jw.

56 or/53-55

57 parent*.mp. or exp Parent-Child Relations/

58 exp Parents/ or parents.mp.

59 famil*.mp. or exp Family/

60 exp Mothers/ or mother*.mp.

61 father*.mp. or exp Fathers/

62 caregiver*.mp. or exp Caregivers/

63 56 or 57 or 58 or 59 or 60 or 61 or 62

64 (m-health or mhealth).mp.

65 mobile health.mp.

66 (smartphone* or smart-phone* or cellphone* or "cell phone" or "cellular phone" or tablet* or ipad or mobile application* or mobile app or mobile apps or phone or iPhone or iphone or android or app or apps or applications or chatbot).mp. [mp=title, abstract, original title, name of substance word, subject heading word, floating sub-heading word, keyword heading word, organism supplementary concept word, protocol supplementary concept word, rare disease supplementary concept word, unique identifier, synonyms]

67 exp Mobile Applications/

68 (gamif* or gamification or game design element* or game-design element* or game element* or game design interface pattern* or game-design interface pattern* or game interface element* or game mechanic* or game feature* or game-like element* or game-like feature* or videogame element* or gamelike or game based or game-based).mp. [mp=title, abstract, original title, name of substance word, subject heading word, floating sub-heading word, keyword heading word, organism supplementary concept word, protocol supplementary concept word, rare disease supplementary concept word, unique identifier, synonyms]

69 64 or 65 or 66 or 67 or 68

70 52 and 63 and 69

71 limit 70 to yr="2008 -Current"

**Embase**

1 (Developmental Disabilit* or Fragile X Syndrome or Learning Disabilit* or dyscalculia or dyslexia or dyslexic or neurodevelopmental disabilit* or Intellectual Disabilit* or Motor Skills Disorder* or Stereotypic Movement Disorder* or Reactive Attachment Disorder* or Child Development Disorder* or Child Behavior Disorder* or "Attention Deficit and Disruptive Behavior Disorder*" or autism or Asperger* or autistic or developmental delay or developmental coordination disorder* or Cerebral Palsy or "attention deficit disorder*" or "Attention Deficit Disorder with Hyperactivity" or ADHD or "attention deficit hyperactive disorder*" or brain injur* or Shaken Baby Syndrome or Traumatic Brain Injur* or Turner Syndrome or mental disorder* or Deglutition Disorder* or Child Behavior Disorder* or Speech Disorder* or Aphasia or Articulation Disorder* or Echolalia or Stuttering or Mutism or motor disability* or motor disorder* or traumatic brain injur* or sensory integration or Down Syndrome or Mental Retardation or Williams Syndrome or sensory disorder* or Hearing Disorder* or hearing loss or Olfaction Disorder* or Somatosensory Disorder* or Taste Disorder* or Vision Disorder* or blindness).mp. [mp=title, abstract, heading word, drug trade name, original title, device manufacturer, drug manufacturer, device trade name, keyword heading word, floating subheading word, candidate term word]

2 exp *developmental disorder/

3 exp *fragile X syndrome/

4 exp *learning disorder/

5 exp *dyscalculia/

6 exp *dyslexia/

7 exp *intellectual impairment/

8 exp *autism/

9 exp *cerebral palsy/

10 exp *attention deficit disorder/

11 exp *brain injury/

12 *mental disease/

13 exp *behavior disorder/

14 exp *speech disorder/

15 exp *Down syndrome/

16 exp *sensory dysfunction/

17 1 or 2 or 3 or 4 or 5 or 6 or 7 or 8 or 9 or 10 or 11 or 12 or 13 or 14 or 15 or 16

18 juvenile/ or exp adolescent/ or exp child/ or exp pediatrics/

19 (child* or pediatric* or paediatric* or prematur* or preterm* or perinat* or neonat* or neo nat* or newborn* or new born* or infan* or baby* or babies or toddler* or boy* or girl* or kid$1 or school* or juvenil* or underage* or under age* or teen* or minor$1 or youth$1 or adolescen* or pubescen* or puberty).mp.

20 (neonat* or infan* or child* or adolescen* or pediatric* or paediatric*).jw.

21 or/18-20

22 (parent* or parents or famil* or mother* or father* or caregiver*).tw,kw.

23 exp *child parent relation/

24 exp *parent/

25 exp *family/

26 exp *mother/

27 exp *father/

28 exp *caregiver/

29 22 or 23 or 24 or 25 or 26 or 27 or 28

30 21 or 29

31 (mHealth or m-health or mobile health or smartphone* or smart-phone* or cellphone* or "cell phone" or "cellular phone" or tablet* or ipad or mobile application* or mobile app or mobile apps or phone or iPhone or iphone or android or app or apps or applications or chatbot).tw,kw.

32 (gamif* or gamification or game design element* or game-design element* or game element* or game design interface pattern* or game-design interface pattern* or game interface element* or game mechanic* or game feature* or game-like element* or game-like feature* or videogame element* or gamelike or game based or game-based).tw,kw.

33 exp *mobile application/

34 31 or 32 or 33

35 17 and 30 and 34

36 limit 35 to yr="2008 -Current"

**PsycInfo**

1 (Developmental Disabilit* or Fragile X Syndrome or Learning Disabilit* or dyscalculia or dyslexia or dyslexic or neurodevelopmental disabilit* or Intellectual Disabilit* or Motor Skills Disorder* or Stereotypic Movement Disorder* or Reactive Attachment Disorder* or Child Development Disorder* or Child Behavior Disorder* or "Attention Deficit and Disruptive Behavior Disorder*" or autism or Asperger* or autistic or developmental delay or developmental coordination disorder* or Cerebral Palsy or "attention deficit disorder*" or "Attention Deficit Disorder with Hyperactivity" or ADHD or "attention deficit hyperactive disorder*" or brain injur* or Shaken Baby Syndrome or Traumatic Brain Injur* or Turner Syndrome or mental disorder* or Deglutition Disorder* or Child Behavior Disorder* or Speech Disorder* or Aphasia or Articulation Disorder* or Echolalia or Stuttering or Mutism or motor disability* or motor disorder* or traumatic brain injur* or sensory integration or Down Syndrome or Mental Retardation or Williams Syndrome or sensory disorder* or Hearing Disorder* or hearing loss or Olfaction Disorder* or Somatosensory Disorder* or Taste Disorder* or Vision Disorder* or blindness).mp. [mp=title, abstract, heading word, table of contents, key concepts, original title, tests & measures, mesh word]

2 exp Developmental Disabilities/

3 exp Fragile X Syndrome/

4 exp Learning Disabilities/

5 exp Acalculia/

6 exp Dyslexia/

7 exp Neurodevelopmental Disorders/

8 exp Intellectual Development Disorder/

9 exp Autism Spectrum Disorders/ or exp Attention Deficit Disorder with Hyperactivity/

10 exp Behavior Disorders/

11 exp Cerebral Palsy/

12 exp Brain Injuries/

13 exp Mental Disorders/

14 exp Speech Disorders/

15 exp Down's Syndrome/

16 exp Williams Syndrome/

17 exp Sensory System Disorders/

18 1 or 2 or 3 or 4 or 5 or 6 or 7 or 8 or 9 or 10 or 11 or 12 or 13 or 14 or 15 or 16 or 17

19 (adolescence 13 17 yrs or childhood birth 12 yrs).ag.

20 (child* or pediatric* or paediatric* or prematur* or preterm* or perinat* or neonat* or neo nat* or newborn* or new born* or infan* or baby* or babies or toddler* or boy* or girl* or kid$1 or school* or juvenil* or underage* or under age* or teen* or minor$1 or youth$1 or adolescen* or pubescen* or puberty).mp.

21 (neonat* or infan* or child* or adolescen* or pediatric* or paediatric*).jw.

22 19 or 20 or 21

23 (parent* or parents or famil* or mother* or father* or caregiver*).mp. [mp=title, abstract, heading word, table of contents, key concepts, original title, tests & measures, mesh word]

24 exp Parent Child Relations/

25 exp Parents/

26 exp Family/

27 exp Mothers/

28 exp Fathers/

29 exp Caregivers/

30 23 or 24 or 25 or 26 or 27 or 28 or 29

31 22 or 30

32 (mHealth or m-health or mobile health or smartphone* or smart-phone* or cellphone* or "cell phone" or "cellular phone" or tablet* or ipad or mobile application* or mobile app or mobile apps or phone or iPhone or iphone or android or app or apps or applications or chatbot).mp. [mp=title, abstract, heading word, table of contents, key concepts, original title, tests & measures, mesh word]

33 (gamif* or gamification or game design element* or game-design element* or game element* or game design interface pattern* or game-design interface pattern* or game interface element* or game mechanic* or game feature* or game-like element* or game-like feature* or videogame element* or gamelike or game based or game-based).mp. [mp=title, abstract, heading word, table of contents, key concepts, original title, tests & measures, mesh word]

34 exp Mobile Applications/

35 32 or 33 or 34

36 18 and 31 and 35

37 limit 36 to yr="2008 -Current"

**ACM digital library:**

AllField:("Developmental Disabilit*" OR "Fragile X Syndrome" OR "Learning Disabilities" OR dyscalculia OR dyslexia OR dyslexic OR "neurodevelopmental disabilit*" OR "Intellectual Disabilities" OR "Motor Skills Disorder" OR "Stereotypic Movement Disorder" OR "Reactive Attachment Disorder" OR "Child Development Disorder" OR "Child Behavior Disorder" OR "Attention Deficit and Disruptive Behavior Disorder" OR autism OR Asperger OR autistic OR "developmental delay" OR "developmental coordination disorder" OR "Cerebral Palsy" OR "attention deficit disorder" OR "Attention Deficit Disorder with Hyperactivity" OR ADHD OR "attention deficit hyperactive disorder" OR "brain injury" OR "Shaken Baby Syndrome" OR "Traumatic Brain Injury" OR "Turner Syndrome" OR "mental disorder" OR "Deglutition Disorder" OR "Child Behavior Disorder" OR "Speech Disorder" OR Aphasia OR "Articulation Disorder" OR Echolalia OR Stuttering OR Mutism OR "motor disability" OR "motor disorder" OR "traumatic brain injury" OR "sensory integration" OR "Down Syndrome" OR "Mental Retardation" OR "Williams Syndrome" OR "sensory disorder" OR "Hearing Disorder" OR "hearing loss" OR "Olfaction Disorder" OR "Somatosensory Disorder" OR "Taste Disorder" OR "Vision Disorder" OR blindness) AND AllField:(child OR children OR adolescent OR adolescence OR youth OR teen* OR "young adult" OR paediatric OR pediatric OR toddler OR infant OR caregiver OR parent OR family OR families OR father OR mother) AND AllField:(mhealth OR m-health OR "mobile health" OR smartphone* OR smart-phone* OR cellphone OR "cell phone" OR "cellular phone" OR tablet* OR ipad OR "mobile application*" OR mobile app OR mobile apps OR phone OR iPhone OR iphone OR android OR app OR apps OR applications OR chatbot OR gamif* OR gamification OR "game design element" OR "game-design element" OR "game element" OR "game design interface pattern" OR "game-design interface pattern" OR "game interface element" OR "game mechanism" OR "game feature" OR "game-like element" OR "game-like feature" OR gamelike OR "game based" OR game-based)

**IEEE Xplore**

"Abstract":"Developmental Disabilit*" OR "Fragile X Syndrome" OR "Learning Disabilities" OR dyscalculia OR dyslexia OR dyslexic OR "neurodevelopmental disabilit*" OR "neurodevelopmental disabilities" OR "Intellectual Disabilities" OR "Motor Skills Disorder" OR "Stereotypic Movement Disorder" OR "Reactive Attachment Disorder" OR "Child Development Disorder" OR "Child Behavior Disorder" OR "Attention Deficit and Disruptive Behavior Disorder" OR autism OR Asperger OR autistic OR "developmental delay" OR "developmental coordination disorder" OR "Cerebral Palsy" OR "attention deficit disorder" OR "Attention Deficit Disorder with Hyperactivity" OR ADHD OR "attention deficit hyperactive disorder" OR "brain injury" OR "Shaken Baby Syndrome" OR "Traumatic Brain Injury" OR "Turner Syndrome" OR "mental disorder" OR "Deglutition Disorder" OR "Child Behavior Disorder" OR "Speech Disorder" OR Aphasia OR "Articulation Disorder" OR Echolalia OR Stuttering OR Mutism OR "motor disability" OR "motor disorder" OR "traumatic brain injury" OR "sensory integration" OR "Down Syndrome" OR "Mental Retardation" OR "Williams Syndrome" OR "sensory disorder" OR "Hearing Disorder" OR "hearing loss" OR "Olfaction Disorder" OR "Somatosensory Disorder" OR "Taste Disorder" OR "Vision Disorder" OR blindness AND "Abstract": child OR children OR adolescent OR adolescence OR youth OR teen* OR "young adult" OR paediatric OR pediatric OR toddler OR infant OR caregiver OR parent OR parents OR family OR families OR father OR mother AND "Abstract" mhealth OR m-health OR "mobile health" OR smartphone* OR smart-phone OR cellphone OR "cell phone" OR "cellular phone" OR tablet* OR ipad OR "mobile application*" OR "mobile app" OR "mobile apps" OR phone OR iPhone OR iphone OR android OR app OR apps OR applications OR chatbot OR gamif* OR gamification OR "game design element" OR "game-design element" OR "game element" OR "game design interface pattern" OR "game-design interface pattern" OR "game interface element" OR "game mechanism" OR "game feature" OR "game-like element" OR "game-like feature" OR gamelike OR "game based" OR game-based
