# Supplementary material for: Gamification in Mobile Apps for Children With Disabilities: Scoping Review
Source: JMIR Serious Games. 2024 Sep 6;12:e49029. doi: 10.2196/49029 (PMC11415723; doi:10.2196/49029)
Supplement: Multimedia Appendix 3 [file games_v12i1e49029_app3.docx]

| **Study characteristics** | | | **Participant Characteristics** | | | **Application characteristics** | | |
| --- | --- | --- | --- | --- | --- | --- | --- | --- |
| **Study, year** | **Country** | **Study design** | **Child diagnosis** | **Sample size** | **Child age (yrs)** | **Cost** | **Device (Mobile, tablets, iPad)** | **Platform (Android, iOS)** |
| Kucirkova et al [46], 2014 | UK | Qualitative case studies | Complex needs and communication problems | 9 | 7-9 | NR | Smartphone, iPad | iOS |
| Moore et al [47], 2015 | Australia | Pilot trial | ASD | 33 parents | <16 | Paid ($25.99AUD) | iPad | iOS |
| Parsons et al [48],  2020 | Australia | Single-site cohort | ASD | 15 parents | NA | Paid ($25.99AUD) | iPad | iOS |
| Parsons et al [49], 2019 | Australia | Exploratory qualitative | ASD | 24 parents | NA | Paid ($25.99AUD) | iPad | iOS |
| Penev et al [50], 2021 | USA | Quasi-experimental feasibility | ASD | 72 | 8.2 | NR | Smartphone | Both |
| Saputra [51], 2016 | Indonesia | Mixed method | Dyslexia | 40 | 5-8 | NR | Smartphone | Android |
| Schmidt et al [52], 2020 | USA | Mixed method | Mild traumatic brain injury | 6 | 11-18 | NR | Smartphone and tablets | NR |
| Thida et al [53], 2020 | Myanmar | usability study | Hearing impairment | 66 | 4-10 | NR | Smartphone | NR |
| Urakami [54], 2021 | Japan | prospective cohort | Physical disability | 60 | 7.2 (3.84) | NR | Smartphone | NR |
| Ying et al [55], 2016 | Malaysia | Qualitative | ASD | 5 | 5-10 | NR | Smartphone and tablets | Android |
| Chua et al [56], 2017 | Singapore | Mixed method (usability) | ASD | 6 | NR | NR | iPad | iOS |
| Doenyas et al [57], 2014 | Turkey | Pilot | ASD | 3 | 4-15 | NR | iPad | iOS |
| Holmes et al [58], 2016 | USA | RCT | Amblyopia | 385 | 5-13 | NR | iPad | iOS |
| Kelly et al [59], 2016 | USA | RCT | Amblyopia | 28 | 4-10 | NR | iPad | iOS |
| Aburukba et al [60], 2017 | UAE | usability study | ASD | 5 | 12-14 | NR | Smartphone | NR |
| Alnaghaimshi et al [61], 2020 | Saudi Arabia | Qualitative (development process of the app) | ASD | NR | NR | NR | Smartphone | Both |
| Barta et al [62], 2017 | Hungary | Qualitative (development process) | ASD | NR | 6-10 | NR | Smartphone | Android |
| Birtwell et al [63], 2019 | USA | Case series | ASD | 1 | 4 | Free | Smartphone and iPads | iOS |
| Borhan et al [64], 2018 | Malaysia | Quasi-experimental | Dyslexia | 8 children, 4 teachers, 2 parents | 7-12 | NR | Smartphone | Android |
| Brkic et al [65], 2022 | UK | Cohort | NDD | 88 | 5-18 | NR | tablets | Android |
| Daud and Abas [66], 2013 | Malaysia | Survey | Dyslexia | 7 dyslexia experts | NA | NR | iPads | iOS |
| Dehkordi and Rias [67], 2014 | Malaysia | Pilot | ASD | 6 | 5-8 | NR | Smartphone | iOS |
| Gómez and Carro [68], 2014 | Spain | Case series | ADHD | 1 girl with ASD and 1 therapist | 9 | NR | Smartphone | NR |
| Guzsvinecz et al [69], 2017 | Hungary | usability | Mild intellectual disability | 50 (20 children with MID) | NR | NR | Smartphone and tablets | Android |
| Hu et al [70], 2019 | Canada | usability | Concussion | 7 children, 7 health care professionals | 10-18 | NR | Smartphone | Android |
| Irwin et al [71], 2015 | USA | quantitative (development process) | ASD | 4 | 8-10 | NR | iPads | iOS |
| Kalantarian et al [72], 2019 | USA | Quasi-experimental | ASD | 8 | 6-10 | NR | Smartphone | Both |
| Macdonald et al [73], 2022 | Canada | Quasi-experimental | ASD | 32 (16 with ASD and 16 with typical development | 3-5 | NR | iPads | iOS |
| Manh et al [74], 2018 | USA | RCT | Amblyopia | 100 | 13-17 | NR | iPads | iOS |
| Mwamba et al [75], 2019 | South Africa | quantitative (testing process) | ADHD | 30 | 5-16 | NR | tablets | Android |
| Tang et al [76], 2021 | UK | Cohort (psychometric properties of the app) | Vision deficiency | 536 | 4-7 | NR | iPads | iOS |
| Cahyono [77], 2022 | Indonesia | Mixed method case-study | Dyslexia | 9 | 7-13 | NR | Smartphone | Android |
| Chistol et al [78], 2023 | Romania | Mixed-method study (development process) | ASD | 60 participants including therapists, teachers and parents | <8 | NR | Smartphone and tablets | Both |
| Tan et al [79], 2023 | Singapore | Delphi method | ASD | 15 parents and 15 experts | 3-12 | Free | Smartphone | Android |
| Schmidt et al [80], 2022 | USA | Mixed methods (Nested cohort of the intervention arm of an RCT) | Mild traumatic brain injury | 36 | 11-18 | NR | Smartphone and tablets | NR |
| Johnson et al [81], 2022 | Australia | Mixed-methods (user-centered design) | Children with neurodevelopmental disabilities | 4 therapists, 3 teachers, 3 parents; 4 children | 6-12 | NR | Smartphone and iPads | iOS |
| Johnson et al [82], 2023 | Australia | Mixed-method feasibility study | Children with neurodevelopmental disabilities | 8 | 6-12 | NR | Smartphone and iPads | iOS |
| Krishnan et al [83], 2021 | India | RCT | ASD | 60 | 13-17 | free | Smartphone and tablets | Android |
