# Supplementary material for: Gamification in Mobile Apps for Children With Disabilities: Scoping Review
Source: JMIR Serious Games. 2024 Sep 6;12:e49029. doi: 10.2196/49029 (PMC11415723; doi:10.2196/49029)
Supplement: Multimedia Appendix 4 [file games_v12i1e49029_app4.docx]

**Multimedia Appendix 4. Reasons for applying gamification found in scoping review**

| Study, year | Purpose | Example Quote |
| --- | --- | --- |
| Penev et al [50], 2021 | **Promote engagement and motivation** | "Developing engaging and effective mobile game-based digital therapeutics requires several sequential steps from ideation to clinical testing and validation  The first step beyond initial concept and storyboarding is co-design with the stakeholders to ensure that players will be sufficiently engaged in the game and to begin to circumscribe the demographics and clinical characteristics of the users for whom the digital therapy can have its highest impact." |
| Saputra [51], 2015 | & **Promote engagement and motivation** | "This research works on the implementation and the effect of gamification approach towards dyslexic learning process. The project, termed LexiPal consists of designing learning model using gamification elements, implementing the design in software application, and measuring the effectiveness of the design toward desired psychological outcomes." |
| Schmidt et al [52], 2020 | **Promote engagement and motivation** | "The SMART application was redesigned with the addition of more interactive and gamified components. Content was also reframed to specifically target  and engage adolescents with mTBI." |
| Chua et al [56], 2017 | **Enhance an intervention’s intended effects**  Enhance learning | "We are interested to develop an iPAD-based game approach for children with ASD to learn emotion through interactive gaming." |
| Doenyas et al [57], 2014 | **Promote engagement and motivation** | "A version of this sequencing game that is more suitable and interesting for older children with autism like Cihan may use more than three cards on each game in order to increase the difficulty of the task and include more than five activities in total in order to keep the player interested." |
| Aburukba et al [60], 2017 | **Promote engagement and motivation** | "The system includes functionalities that allows Teachers to customize the games and set them as an assignment for the child to play with, according to the pre-defined selections. In the event of a teacher has to assign a game, the teacher can select a level of the respective game and different customizations." |
| Barta et al [62], 2017 | **Promote engagement and motivation** | "This part gives a new platform for playing, winning, achieving success. This  motivation of the children is of great importance; a rewards page is added to both parts. Thus, both the completed tasks and the practice are rewarded." |
| Birtwell et al [63], 2019 | **Enhance an intervention’s intended effects**  Enhance learning | "… uses the individual’s restricted interests to teach skills and enhance communication within one interactive mobile app. Therefore, Sidekicks! offers an innovative mobile intervention platform that can specifically tap into the individuals’ highly preferred interests, thereby enhancing motivation." |
| Borhan et al [64], 2018 | **Enhance an intervention’s intended effects**  Enhance learning | "Multimedia elements are integrated into the mobile application to make the learning environment more attractive and interactive for this special group of children." |
| Brkic et al [65], 2022 | **Promote engagement and motivation** | "In short, FarmApp is an age- and developmental-stage appropriate, intuitive, non-verbal cognitive assessment platform, with a game-like structure, i.e., motivating and enjoyable for participants." |
| Daud and Abad [66], 2013 | **Enhance an intervention’s intended effects**  Intervention efficacy | "Dyslexia Baca provides visual graphics of confusing letters as an errorless game for dyslexic children to play. This step can assist them to differentiate the dissimilarities between letters. The main objective is to help dyslexic children to recognize and distinguish letters p, q, b, d, m." |
| Tang et al [76], 2021 | **Enhance an intervention’s intended effects**  Intervention efficacy | "Secondly, gamification with a fun, intuitive and professionally animated interface helps children to maintain engagement and complete the test." |
| Cahyono [77], 2022 | **Promote engagement and motivation** | "dyslexic children are much less motivated than non-dyslexic students in reading... In order to increase the motivation of dyslexic students, it is suggested to employ motivational teaching techniques. Therefore, this research explores how gamification advancement fosters learning motivation." |
| Schmidt et al [80], 2022 | **Promote engagement and motivation** | "Participants received real-time, patient-centric symptom and activity feedback reports to encourage self-management and augment ongoing care." |
| Johnson et al [81], 2022 | **Enhance an intervention’s intended effects**  **Improve Adherence** | " The gamification strategy for this app was designed with consideration of the context: supporting children in a classroom or home environment to complete nondigital therapy activities and exercises." |
| Johnson et al [82], 2023 | **Enhance an intervention’s intended effects**  **Improve Adherence** | ""This research informed the development of a new purpose-driven app, called ‘Zingo’, designed to deliver home and school therapy programs in a way that is fun and effective in improving adherence for children" |
