# Supplementary material for: Gamification in Mobile Apps for Children With Disabilities: Scoping Review
Source: JMIR Serious Games. 2024 Sep 6;12:e49029. doi: 10.2196/49029 (PMC11415723; doi:10.2196/49029)
Supplement: Multimedia Appendix 5 [file games_v12i1e49029_app5.docx]

## **Multimedia Appendix 5.** Included studies with results directly related to an intervention **(n = 15)**

| **Study, year** | **Country** | **Study Design** | **Sample description: size N=, Age (yrs), disability** | **Time point data was collected** | **Intervention (name of app)** | **Control or comparison group** | **Findings/outcomes related to intervention** |
| --- | --- | --- | --- | --- | --- | --- | --- |
| **Quantitative** |  |  |  |  |  |  |  |
| Parsons et al [48], 2020 | Australia | Single-site cohort | **Population:** parents of children with autism under 16 years old  **N:** 15 | baseline (T1), post- intervention (T2) and follow-up at 12 months  post-intervention (T3) using  1) Mullen Scales of Early Learning (MSEL)  2) Symbolic Play Test (SPT) and  3)Communication and Symbolic Behavior Scale | iPad app (TOBY) | No | Receptive language, social skills, pragmatic language and playfulness of children with autism spectrum disorder improved during the three-month intervention period and were maintained at least 12 months after using the app. |
| Penev et al [50], 2021 | USA | Quasi-experimental | **Population:** children (75% male, average age 8 years 2 months) with autism  **N:** 72 | Baseline and after using the app for 4 weeks | mobile app (GuessWhat) | No | significant improvements in Social Responsiveness Score-2 (SRS-2) total (3.97, p <0.001) and Vineland Adaptive Behavior Scales-II (VABS-II) socialization standard (5.27, p=0.002) scores. |
| Urakami [54], 2021 | Japan | Prospective cohort | **Population**: children with short stature  **N**: 60 | Number of injections over a 24-week period | Mobile app (GROWJECTOR) | No | Median and mean adherence rates after 24 weeks of observation  were 96% and 93%, respectively.  However, adherence rates were lower from week 16 to week 20 compared to week 1 to Week 4. |
| Doenyas et al [57], 2014 | Turkey | Quasi-experimental | **Population**: 4-15 children with autism  **N**: 3 | Data collection over 2-week period | iPad app | No | The study has mixed results in teaching sequencing skills to children with autism as they have different age ranges. The app was easy enough for the 15-year-old child and could not help the 4-year-old child. On the other hand, it could improve the sequencing skills of the 11-year-old child. *More individualization is needed.* |
| Holmes et al [58], 2016 | USA | RCT | **Population**: children with Amblyopia  **N**: 190 intervention group, and 195 control group | amblyopic-eye visual acuity from baseline to 16 weeks | iPad app (PEDIG) | Yes; patching  Meaning: placing a patch **(occlusion or penalization**) over the better-seeing eye to treat amblyopia.) | " In younger participants (aged 5 to <7 years) without prior amblyopia treatment, amblyopic-eye VA improved by a mean (SD) of 2.5 (1.5) lines in the binocular group and 2.8 (0.8) lines in the patching group."  VA improvement in the intervention group was not as good as with 2 hours of prescribed daily patching (control group). |
| Kelly et al [59], 2016 | USA | RCT | **Population**: children with Amblyopia  **N**: 14 intervention group, and 14 control group | Baseline and 4 weeks | iPad app (Dig Rush) | Yes; patching | "A binocular iPad game was effective in treating childhood  amblyopia and was more efficacious than patching at the 2-week visit." |
| Borhan et al [64], 2018 | Malaysia | Quasi-experimental | **Population**: 8 children with dyslexia, 4 teachers, 2 parents | Pre and post intervention; using questionnaire at the end of the intervention | Mr. Read mobile app | No | The use of Mr. Read V2.0 led to a 28% improvement in children's test scores. All respondents, including instructors, parents, and children, agreed or strongly agreed that the mobile application enhances reading skills. The sight word reading strategy incorporated into the app is particularly effective for helping dyslexic children improve their reading abilities. |
| Kalantarian et al [72], 2019 | USA | Quasi-experimental | **Population**: 6-10 children with autism  **N**: 8 | During the lab trial | Mobile app (Guesswhat) | No | The results of the study showed that the gamified mobile platform is effective in creating highly emotive structured videos. It identifies the most effective prompts and categories for eliciting a diverse range of emotions in participants. |
| Macdonald et al [73], 2022 | Canada | Quasi-experimental | **Population**: 32 (16 with ASD and 16 with typical development **N**: 32 | Pre and post intervention | iPad app (Not reported) | No | The findings revealed a significant increase in reading comprehension scores. |
| Manh et al [74], 2018 | USA | RCT | **Population**: 13-17 years old children with Amblyopia  **N**: 100 | Pre and post intervention | iPad app (not reported) | Yes; part-time patching | The mean visual acuity (VA) improvement in the amblyopic eye was 3.5 letters (95% CI: 1.3-5.7 letters) for the binocular group and 6.5 letters (95% CI: 4.4-8.5 letters) for the patching group. After adjusting for baseline VA, the difference between the groups was -2.7 letters (95% CI: -5.7 to 0.3 letters, P = .082), favoring the patching group by about 0.5 lines. In the binocular group, only 13% of participants completed more than 75% of the prescribed treatment according to iPad adherence data.  For teenagers aged 13 to under 17 years, the binocular iPad game did not result in better improvement in amblyopic eye VA compared to patching and may be less effective. |
| Krishnan et al [83], 2021 | India | RCT | **Population:** 13- to 17-year-old school going adolescent with autism  **N:** 30 intervention group, 30 control group | Plaque Index and gingival index were assessed at baseline, 6th week and after 12th week. | Mobile app (Brush Up) | Yes: visual cards | A significant difference in plaque (p = < 0.001) and gingival scores (p = < 0.001) were seen among the groups after 6- and 12-weeks post-intervention. However, there was no statistically significant difference in dental plaque and gingival scores between the groups at all the timelines. |
| **Qualitative** |  |  |  |  |  |  |  |
| Kucirkova et al [46], 2014 | UK | Qualitative case studies | **Population:** 7-9 years old children with Complex needs and communication problems  **N:** 9 | Observations over a 7-week period | iPad app (Our story) | No | The case studies shows that this app can (a) be highly motivating for children with a range of language and socio-emotional difficulties and (b) contribute to their story-sharing and story-creation abilities. However, the extent to which a child can use the app to create and shares a story heavily depends on the severity of a child's disability. |
| Parsons et al [49], 2019 | Australia | Exploratory qualitative | **Population:** parents of children with autism  **N:** 24 | Interviews after a 3-month trial | iPad app (TOBY) | No | Parents expressed that the app could provide benefits for them and their children by improving behaviour, visual-spatial, fine motor skills etc., *however, they highlighted that the app were not fully successful in providing choice and considering the individuality.* |
| **Mixed methods** |  |  |  |  |  |  |  |
| Cahyono [77], 2022 | Indonesia | Mixed method case-study | **Population:** 7-13 years old children with dyslexia, and 3 teachers  **N:** 9 children and 3 teachers | Questionnaire at the end of the intervention to access children's perceptions; semi-structured interviews with teachers | Mobile app (LexiPal) | No | The findings indicate positive trends in the usability of LexiPal’s gamification features, enhancing both intrinsic and extrinsic motivation. Intrinsic motivation is driven by elements such as levelling and the app’s interface, while extrinsic motivation is supported by reward and praise mechanisms. Additionally, teachers observed cognitive improvements in pupils using LexiPal, specifically in fine motor skills, emotion comprehension, time and direction comprehension, and sentence writing. |
| Schmidt et al [80], 2022 | USA | Mixed-methods (Nested cohort of the intervention arm of an RCT) | **Population**: 11-18 years old children with Mild traumatic brain injury  **N**: 36 | Automatic app usage during the trial; several questionnaires including Connor-Davidson Resilience Scale, Coping Strategies Inventory-short form, Post Concussion Symptom Scale (PCSS) were administered at the baseline; qualitative feedback was collected at the end of the study. | Mobile app (SMART) | No | Adolescents of higher socioeconomic status and those who manage their emotions using active engagement spent more time on both components of the SMART program. Qualitative feedback indicates that some users and parents were hesitant to use their phones for symptom monitoring and receiving psychoeducation shortly after an injury. They expressed a preference for paper and pencil methods instead. |
